# Hypercapnia attenuates ventilator-induced lung injury through vagus nerve activation^1^

**DOI:** 10.1590/s0102-865020190090000002

**Published:** 2019-11-25

**Authors:** Wenfang Xia, Guang Li, Zhou Pan, Qingshan Zhou

**Affiliations:** I MD, Department of Critical Care Medicine, Renmin Hospital of Wuhan University, Wuhan, Hubei, China. Conception of the study, analysis of data, manuscript writing, critical revision; II MD, Department of Critical Care Medicine, Renmin Hospital of Wuhan University, Wuhan, Hubei, China. Technical procedures, critical revision; III MD, Department of Critical Care Medicine, Renmin Hospital of Wuhan University, Wuhan, Hubei, China. Conception of the study, analysis of data, critical revision

**Keywords:** Hypercapnia, Ventilator-Induced Lung Injury, Vagus Nerve, Rats

## Abstract

**Purpose:**

To investigate the role of vagus nerve activation in the protective effects of hypercapnia in ventilator-induced lung injury (VILI) rats.

**Methods:**

Male Sprague-Dawley rats were randomized to either high-tidal volume or low-tidal volume ventilation (control) and monitored for 4h. The high-tidal volume group was further divided into either a vagotomy or sham-operated group and each surgery group was further divided into two subgroups: normocapnia and hypercapnia. Injuries were assessed hourly through hemodynamics, respiratory mechanics and gas exchange. Protein concentration, cell count and cytokines (TNF-α and IL-8) in bronchoalveolar lavage fluid (BALF), lung wet-to-dry weight and pathological changes were examined. Vagus nerve activity was recorded for 1h.

**Results:**

Compared to the control group, injurious ventilation resulted in a decrease in PaO_2_/FiO_2_ and greater lung static compliance, MPO activity, enhanced BALF cytokines, protein concentration, cell count, and histology injury score. Conversely, hypercapnia significantly improved VILI by decreasing the above injury parameters. However, vagotomy abolished the protective effect of hypercapnia on VILI. In addition, hypercapnia enhanced efferent vagus nerve activity compared to normocapnia.

**Conclusion:**

These results indicate that the vagus nerve plays an important role in mediating the anti-inflammatory effect of hypercapnia on VILI.

## Introduction

Mechanical ventilation (MV) is the primary means of treating acute respiratory distress syndrome (ARDS)^1^. MV improves airway injury and oxygenation, but can cause or aggravate lung injury leading to ventilator-induced lung injury (VILI)^2^. Lung protection ventilation strategies such as reducing tidal volume and increasing positive end-expiratory pressure (PEEP) levels can increase the survival rate in ARDS patients^3^. However, these measures can lead to elevated PaCO_2_, resulting in hypercapnia (HPC) which has previously been considered an adverse side effect of lung protection ventilation strategies. However, recent studies have shown that HPC can have a protective effect on multiple organs, and that the application of hypercapnia can improve ALI/ARDSs^4^. In addition, hypercapnic acidosis can directly attenuate experimental acute lung injuries induced by ischemia-reperfusion^5^, free radicals^6^, endotoxins^7^, systemic sepsis^8^, and VILI both ex vivo and in vivo^9^. These studies indicate that hypercapnic acidosis may reduce lung injury through the inhibition of the nuclear factor-κB inhibitor IκBα and the reduction of cytokines through anti-inflammatory mechanisms^10^.

The cholinergic anti-inflammatory pathway (CAP), a newly discovered neuro-regulatory pathway, can effectively reduce the release of a variety of pro-inflammatory factors, leading to a reduction in systemic and local inflammation. The anti-inflammatory effects of the CAP are dependent on the activation of the vagus nerve. Animal experiments have shown that vagotomy increases inflammatory injury, leading to greater vulnerability to the attack of inflammatory stimulation. Borovikova *et al*.^11^ reported that vagotomy enhances inflammation and mortality in experimental animals. Wolfram *et al*.^12^ found that after colon ascendens stent peritonitis surgery, vagotomy led to significantly increased mortality compared to sham-vagotomized animals. In addition, it has been shown that vagotomy results in enhanced severity of pancreatitis, as reflected by histology, edema, plasma hydrolases, and interleukin-6 levels^13^, and increased infiltration of peritoneal neutrophil granulocytes and macrophages, ultimately leading to an increase in mortality. In addition, vagotomy may influence the recruitment and activity of immune cells to participate in inflammation. Mihaylova *et al*.^14^ found that vagotomy leads to a dramatic decline in immune cell counts (including CD4C T cells and CD8C T cells) in the septic spleen.

In the current study, we hypothesized that hypercapnia plays an important role in the protection of VILI through the activation of the vagus nerve. The aim of the present study was to test whether treatment with hypercapnia could activate vagus nerve activity and attenuate VILI.

## Methods

All animal procedures adhered to the Guide for the Care and Use of Laboratory Animals, and were performed in accordance with the ethics committee of the Wuhan University.

Specific-pathogen-free adult male Sprague-Dawley rats weighing between 200 and 250 g were maintained in standardized housing with a 12:12h dark light cycle with free access to water and food. Rats were anaesthetized by intraperitoneal injection of pentobarbital (40 mg/kg) and xylazine (10 mg/kg). After tracheotomy, a metal cannula was inserted into the trachea and connected to a ventilator (Model 683 Ventilator, Harvard Apparatus, Kent, United Kingdom). Mechanical ventilation was set to a tidal volume (Vt) of 8 ml/kg, an inspired oxygen fraction (FiO_2_) of 0.50, a respiratory rate of 60 breaths/min and a positive end-expiratory pressure (PEEP) of 0 cm H_2_O for the first 10 mins. The Vt was then increased to 30ml/kg to create high-stretch ventilation in the experimental groups, and these conditions were maintained until the end of the experiment. The respiratory rate was decreased to 12 beats/min in order to maintain a constant minute ventilation and physiologic blood pH. The right cervical vagus nerves were dissected and exposed for vagotomy or discharge recording. The left carotid artery was catheterized for arterial blood pressure (ABP) measurement and arterial blood gas analysis.

### Experimental protocol

**Figure 1:**
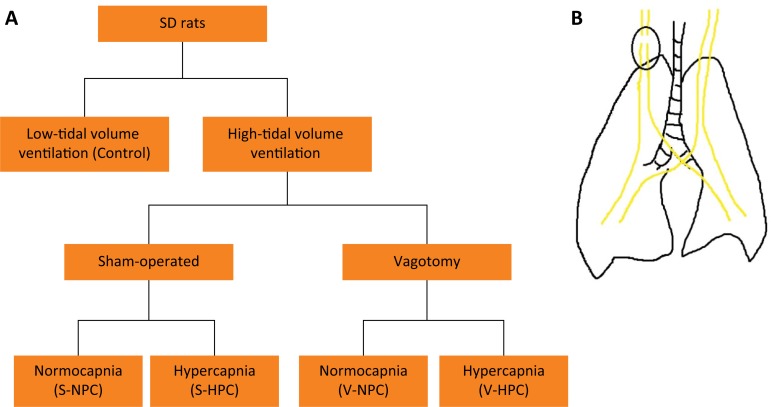
**(A)** Schematic flow chart of the study design. **(B)** Vagotomy illustration. A ventral cervical midline incision was made to expose the right vagal trunk, which was separated from the carotid artery and then transected.

Control rats (n = 8) were maintained at a low Vt ventilation as described above (Vt = 8 ml/kg, PEEP = 0 cm H_2_O, FiO_2_ = 0.5). The VILI groups (n = 32) were randomly assigned to either vagotomy (n = 16) or sham-operated group (n = 16) and each group was further divided into two subgroups: normocapnia (FiCO_2_=0) or hypercapnia (FiCO_2_=0.5) for a period of 4 h (n = 8 per/group). There were thus, four VILI groups: V-NPC (n = 8), V-HPC (n = 8), S-NPC (n = 8) and S-HPC (n = 8) (Fig. 1).

Hemodynamics, lung compliance and arterial blood gas measurements were performed hourly throughout the experiment. After 4 h of mechanical ventilation, all rats were sacrificed with a high dose of pentobarbital (100 mg/kg, i.v.). The chest was opened and the lungs were rapidly removed and the right lung was rinsed three times with a single 5 ml aliquot of 4°C fresh saline with fluid recovery of approximately 85–90%. The obtained fluid was centrifuged (1.500 rpm for 10 min at 4°C), and the supernatant was immediately frozen on dry ice and stored at −80°C until processing. The left lung was used for the measurement of wet-to-dry ratio for the assessment of edema. The right upper lung tissues were stored in 4% paraformaldehyde for pathological analysis and myeloperoxidase (MPO) examination.

### Vagotomy and vagus nerve activity

Vagotomy was performed by severing the right cervical vagal nerve after it was separated from the carotid sheath. The incision was then sutured closed. The cervical vagal nerve in the sham-operated group was isolated from surrounding tissue but not transected. All procedures followed aseptic techniques and repeated doses of pentobarbital were infused as necessary throughout the operation. In the vagotomized rats, the proximal end of the right vagus nerve was placed on bipolar platinum electrodes (Plastics One, Roanoke, VA) and covered with mineral oil. The sampling signal rate was at 1 KHz and signals were passed through a band-pass filter (100–1000 Hz). Nerve activity was measured using the BL-420F data acquisition and analysis system (Chengdu Taimeng Software Co.LTD, China) during the last 60 min of the experiment.

### Histological analysis

After sacrifice, right upper lung tissue was fixed in 4% paraformaldehyde solution for 24h, dehydrated in graded ethanol concentrations, hyalinization and embedded in paraffin. Tissue was sectioned at a thickness of 4 μm and stained with hematoxylin and eosin according to standard methods. Lung injury was scored on a scale of 0–4 based on the average of the following items: (I) alveolar capillary congestion; (II) hemorrhage; (III) infiltration of neutrophils into the airspace or the vessel wall; and (IV) alveolar wall thickness/hyaline membrane formation^15^. A score of 0 signified normal findings, while scores of 1–4 represented mild (<25%), moderate (25–50%), severe (50–75%), and very severe (>75%) lung injury, respectively.

### Physiologic measurements

In all experimental series, systemic mean blood pressure (MAP) and heart rate (HR) were recorded at baseline, initiation of test conditions, and at 1-hour intervals thereafter. The blood gas parameters of pH, PO_2_, and PCO_2_ were measured hourly with an i-STAT Blood Gas Analyzer (Abbot, Hoofddorp, the Netherlands).

### Lung wet-to-dry weight ratio

Lung wet-to-dry weight ratio was used as a measure of pulmonary edema. The fresh left lung was weighed immediately after collection and placed into a 60°C oven to dry for 72h. The dried tissue was then weighed to determine the wet-to dry weight ratio.

### Lung mechanics assessment

Airway plateau pressure was measured while holding the breath for 4 seconds at the end inspiratory phase. Lung elastance was calculated as (plateau pressure- PEEP)/Vt. After 4h of mechanical ventilation, lung static compliance was measured using pressure-volume curve.

### Myeloperoxidase assay

The inferior lobe of the left lung was stored at −80°C until processing. The tissue was ground into a homogenate to measure MPO activity using a kit (Jiancheng Bio-Technology, Nanjing, China) according to the manufacturer's protocol. Briefly, frozen lung specimens were weighed and homogenized in hexadecyltrimethylammonium bromide (HTAB) buffer (0.5% HTAB in 50 mM phosphate buffer; pH 6.0). Each sample was then sonicated and centrifuged at 40,000 × g for 15 min. MPO activity in each supernatant was assayed to determine the extent of H_2_O_2_-dependent oxidation of *ο*-dianisidinehydrochloride. Absorbance values were measured on a spectrophotometer at 460 nm and were recorded. MPO activity per gram of lung weight was calculated for each sample.

### Bronchoalveolar Lavage Fluid (BALF) protein and total cell counting

BALF was collected by opening the chest via sternotomy. The right main stem bronchus was clamped with a hemostat, the trachea was cannulated and bronchoalveolar lavage (BAL) of the right lung was performed by flushing the lung and airways three times with 5 ml cold (4°C) saline solution and 4 ml of BALF was collected. A 1-ml aliquot of BALF was used for cell counts. The remaining fluid was centrifuged (300 × g at 4°C for 10 min) and the cell-free supernatant was divided into two 1-ml aliquots. One aliquot was snap-frozen in liquid nitrogen and stored at −80°C for subsequent analysis of tumor necrosis factor (TNF-α) and interleukin-8 (IL-8), using commercial enzyme-linked immunosorbent assay kits (R&D Systems, Minneapolis, MN, USA). The remaining aliquot was frozen at −20°C for a measurement of total protein concentration (BCA; Pierce, Rockford, IL).

### Statistical analysis

Statistical analysis was carried out using SPSS 17.0 software (SPSS Inc, Chicago, IL, USA). A one- or two-way analysis of variance (ANOVA) or a Student's t-test was used for data analysis. When appropriate, group differences were explored using a Bonferroni post hoc test, or a Mann-Whitney nonparametric test.

## Results

### Hemodynamic variables and gas exchange

MAP and HR remained stable throughout the four hours of mechanical ventilation in all groups. The HR in the S-HPC group tended to be higher than the control group, but the difference did not reach statistical significance. The results of the blood gas analyses (PaO_2_, PaCO_2_ and pH values) are shown in Table 1. In the control group, the PaO_2_/FiO_2_ remained stable from 422 ± 32 mmHg to 414 ± 16 mmHg. However, the PaO_2_/FiO_2_ dropped steadily from 428 ± 28 mmHg to 308 ± 31 mmHg (*P*<0.001) in the S-NPC group while the PaO_2_/FiO_2_ were stable and significantly increased at each time point in the S-HPC group compared to the S-NPC group (*P*<0.05). There were no significant differences in PaO_2_/FiO_2_ between the HPC and NPC vagotomized rats. Due to metabolic acidosis, the hypercapnic groups (S-HPC and V-HPC group) had significantly lower pH values at every time point. However, pH values did not significantly change in the normocapnia groups.

**Table 1:** Hemodynamic parameters and gas exchange.

	Baseline	1h	2h	3h	4h
MAP (mmHg)					
Control	132 ± 10	128 ± 9	124 ± 8	128 ± 6	130 ± 9
V-HPC	128 ± 8	126 ± 9	122 ± 7	124 ± 6	138 ± 10
V-NPC	118 ± 6	116 ± 8	114 ± 7	122 ± 8	125 ± 9
S-HPC	126 ± 7	122 ± 8	124 ± 11	121 ± 10	125 ± 8
S-NPC	122 ± 9	128 ± 10	126 ± 8	122 ± 8	132 ± 8
HR					
Control	325 ± 16	308 ± 14	330 ± 12	318 ± 14	317 ± 12
V-HPC	320 ± 14	318 ± 22	320 ± 17	330 ± 16	314 ± 18
V-NPC	318 ± 16	336 ± 14	330 ± 14	330 ± 18	340 ± 22
S-HPC	322 ± 18	318 ± 20	321 ± 16	330 ± 14	306 ± 24
S-NPC	318 ± 14	340 ± 22	338 ± 14	337 ± 15	336 ± 26
PaO_2_/FiO_2_(mmHg)					
Control	422 ± 32	416 ± 24	398 ± 14	408 ± 12	414 ± 16
V-HPC	428 ± 28	316 ± 18^a^	308 ± 14^a^	304 ± 17^a^	308 ± 31^a^
V-NPC	422 ± 31	350 ± 16^a^	320 ± 18^a^	290 ± 16^a^	280 ± 12^a^,^b^
S-HPC	420 ± 30	410 ± 25^c^	390 ± 20^c^	400 ± 21^c^	408 ± 30^c^
S-NPC	418 ± 25	340 ± 18^a^	310 ± 19^a^	308 ± 19^a^	320 ± 16^a^
pH					
Control	7.40 ± 0.07	7.38 ± 0.06	7.36 ± 0.04	7.36 ± 0.06	7.40 ± 0.08
V-HPC	7.45 ± 0.06	7.15 ± 0.08^a^	7.10 ± 0.07^a^	7.01 ± 0.04^a^	6.99 ± 0.09^a^
V-NPC	7.38 ± 0.09	7.30 ± 0.06^b^	7.28 ± 0.04^b^	7.34 ± 0.07^b^	7.32 ± 0.07^a^,^b^
S-HPC	7.36 ± 0.12	7.12 ± 0.06^c^	7.10 ± 0.08^a^,^c^	7.06 ± 0.05^a^,^c^	7.01 ± 0.08^a^,^c^
S-NPC	7.40 ± 0.08	7.35 ± 0.08	7.38 ± 0.08	7.40 ± 0.04	7.38 ± 0.01

Values are means ± SD; n = 8/group.

a *P* <0.05 *vs*. baseline;

b *P* <0.05 *vs* V-HPC;

c *P*<0.05 *vs*. S-NPC.

### Vagus nerve activity

**Figure 2:**
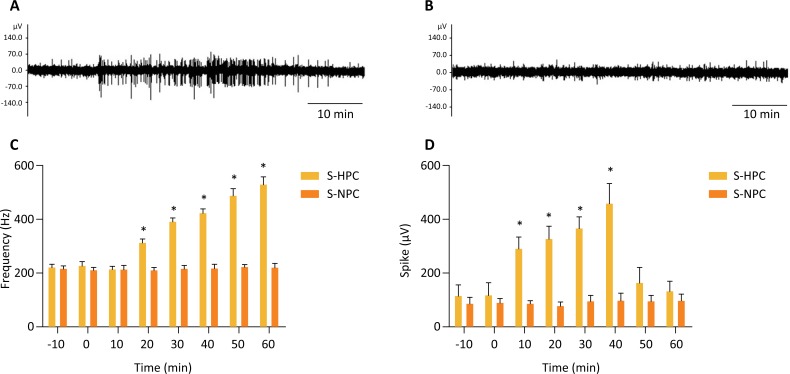
**(A)** The effect of hypercapnia on discharge activity of the cervical vagus nerve. **(B)** The effect of normocapnia on discharge activity of the cervical vagus nerve **(C and D)**. The frequency and spike activity of the cervical vagus nerve. Values are means ± SD; n = 8/group. *P<0.05 *vs*. baseline.

Discharge frequency from the vagus nerve was significantly increased after 20 min of hypercapnia when compared to baseline or normocapnia (Fig. 2 A–B). The increase in discharge continued for one hour. In addition, spike activity was significantly enhanced starting 10 min after hypercapnia (Fig. 2 C–D).

### Pathological changes

**Figure 3:**
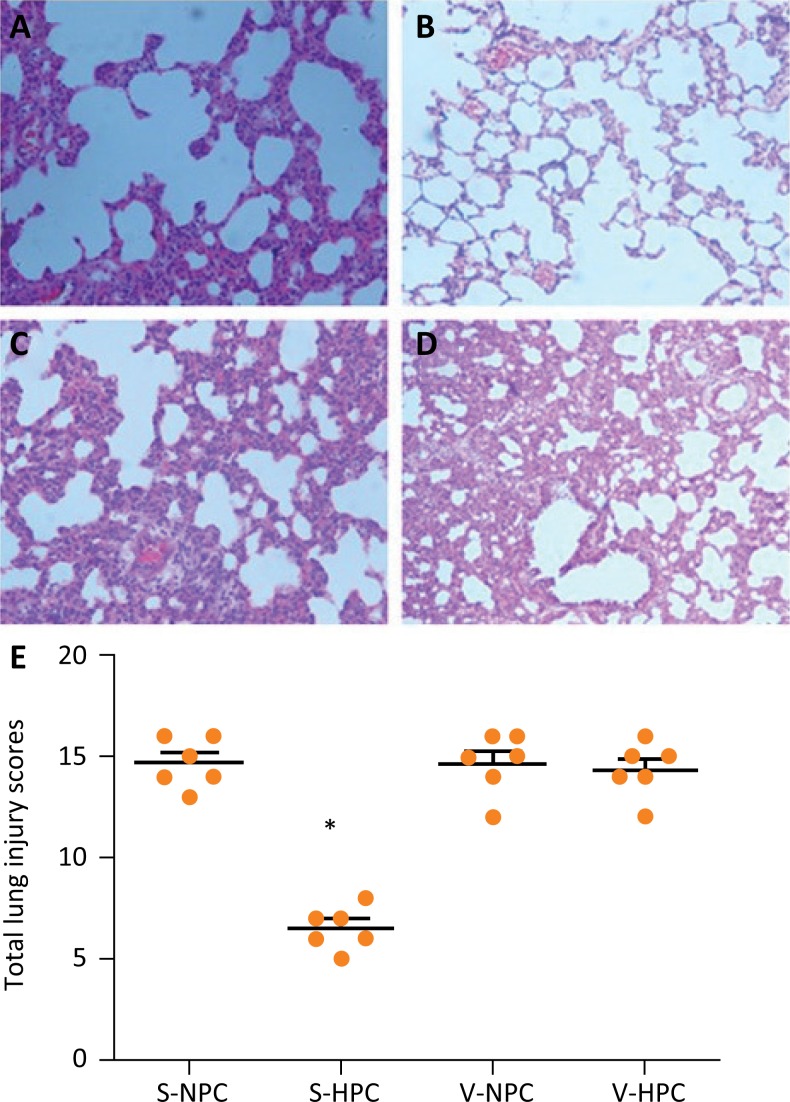
Histological analysis of lung injury. **(A)** S-NPC group, **(B)** S-HPC group, **(C)** V-NPC group, **(D)** V-HPC group, and **(E)** Lung injury scores for each group. Horizontal bars represent the median, n = 8/group. *P<0.05 *vs*. the control group, ^#^ *P*<0.05 *vs*. the S-NPC group.

Compared to the control group, tissue from the VILI groups showed a thickened alveolar septum and edema, and inflammatory cell and neutrophil infiltration into the pulmonary tissue (Fig. 3 A–B). Treatment with HPC lead to decreased pulmonary edema, thinning of the alveolar septum, lower infiltration of inflammatory cells, and decreased exudation of neutrophils (Fig. 3C) and ultimately, a lower lung injury score (Fig. 3E). However, vagotomy abolished the protective effects of HPC on VILI rats, as seen by thickened alveolar septum and edema, and inflammatory cell and neutrophil infiltration into the pulmonary tissues (Fig. 3D). Analysis of the lung injury scores demonstrated significantly greater injury scores in the S-NPC group vs the control group (*P*<0.001). Lung injury score was significantly attenuated in VILI rats treated with hypercapnia (the S-HPC group). However, hypercapnia treatment of vagotomized VILI rats (V-HPC group), did not significantly alter lung injury score (no significant difference between the V-HPC group and the V-NPC group).

### Lung mechanics

**Figure 4:**
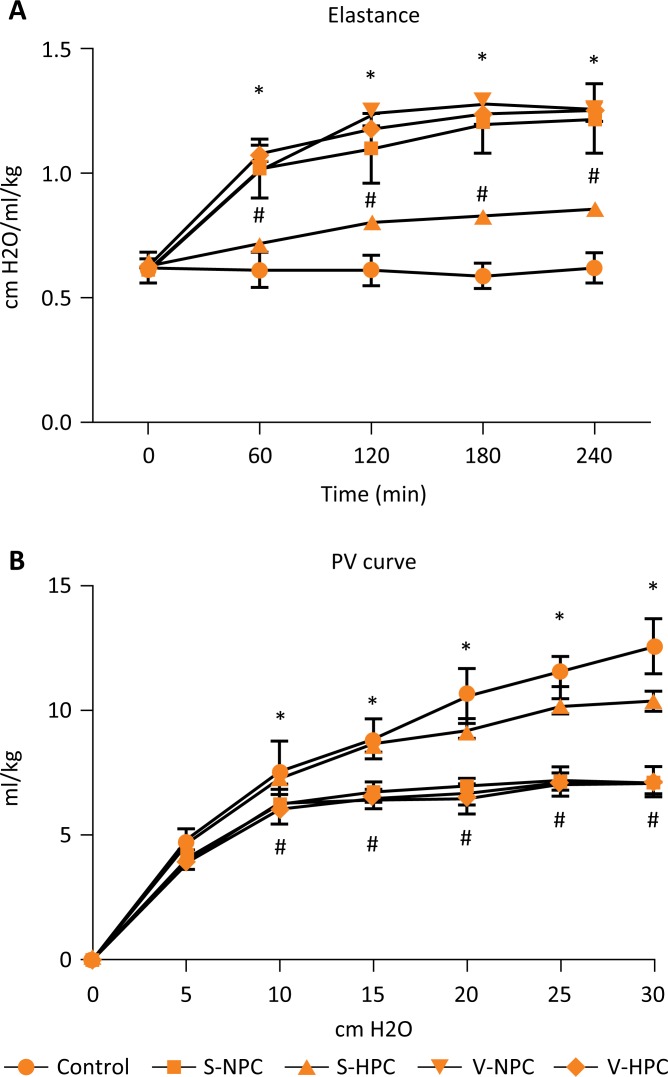
**(A)** Lung elastance changes during 4 hours of mechanical ventilation. **(B)** Static compliance curves at the end of the four-hour mechanical ventilation. Values are means ± SD; n = 8/group. *P<0.05 *vs*. the control group. ^#^ *P*<0.05 *vs*. the S-NPC group.

Lung elastance values at baseline were similar in all groups. However, after 60 minutes, lung elastance increased significantly in all groups compared to the control group (Fig. 4A). Hypercapnia treatment in the sham group (S-HPC) significantly improved elastance. However, hypercapnia in the vagotomy group (V-HPC) did not lead to improvement. Similarly, the static pressure–volume curves showed that all groups had lower compliance compared with the control group, (Fig. 4B). However, the pressure-volume relationship of the S-HPC group shifted to the left, while the pressure-volume relationship of the V-HPC group, did not significantly differ from the V-NPC group.

### Wet-to-dry weight ratio

The wet-to-dry weight ratio was significantly increased in VILI rats compared to the control group, indicating increased lung edema (Table 2). The wet-to-dry ratio was significantly reduced in the S-HPC group compared to the S-NPC group (3.1 ± 0.4 *vs*. 5.3 ± 0.2, *P*<0.05), while hypercapnia had no significant effect on wet-to-dry weight ratio in vagotomized rats (V-HPC *vs*. V-NPC; 5.1 ± 0.3 *vs*. 4.9 ± 0.2, respectively; *P*> 0.05).

**Table 2:** Comparison of the protein level, cell counts, TNF-α and IL-8 in BALF; MPO activity and W/D ratio in lung tissue.

	Control	S-NPC	S-HPC	V-NPC	V-HPC
TNF-α (pg/ml)	98 ± 6	364 ± 14*	102 ± 23#	386 ± 26*	420 ± 35*
IL-8 (pg/ml)	440 ± 28	1.164 ± 44*	540 ± 45#	1.048 ± 38*	1.254 ± 46*
Protein (g/L)	0.78 ± 0.03	2.6 ± 0.4*	0.8 ± 0.04#	2.8 ± 0.6*	2.8 ± 0.4*
Cell Count x10^7^	4.9 ± 0.8	18.6 ± 3.2*	5.2 ± 1.6#	20.4 ± 4.2*	19.8 ± 3.6*
MPO (U/g)	1.4 ± 0.3	4.4 ± 0.4*	1.6 ± 0.2#	4.3 ± 0.5*	4.2 ± 0.8*
Wet-to-Dry	2.8 ± 0.3	5.3 ± 0.2*	3.1 ± 0.4#	5.1 ± 0.3*	4.9 ± 0.2*

Values are means ± SD; n = 8/group.

* *P*< 0.05 *vs*. Control;

# *P*< 0.05 *vs*. S-NPC.

### BALF and MPO Activity in Lung Tissue

The BALF total cell count, protein concentration, MPO activity, and levels of TNF-α and IL-8 were all increased in VILI rats compared to control rats (Table 2). Treatment with hypercapnia (S-HPC group) significantly reduced cell count, protein concentration, MPO activity and levels of TNF-α and IL-8 in sham operated rats. However, in vagotomized rats, hypercapnia (V-HPC) did not significantly decrease any measure (Table 2).

## Discussion

The main findings of this study are as follows: (1) Injurious MV for 4 h is associated with pulmonary edema, increased levels of BALF inflammatory cytokines TNF-α and IL-8, neutrophil counts, MPO activity, and structural damage. (2) Therapeutic hypercapnia retained the lung wet-to-dry weight ratio, BALF protein content, TNF-α and IL-8 levels, and lung injury score to control level. (3) Vagotomy after VILI abolished the protective effects of hypercapnia. Our data suggest that hypercapnia plays an important role in the protection of VILI through the activation of the vagus nerve.

In the present study, VILI was induced by high volume ventilation. We found that 5% fraction of inhaled CO_2_ in experimental animals could maintain PaCO_2_ between 60-80 mmHg. This method allows for increasing PaCO_2_ without changes in RR or VT. Previous animal studies have found that inhaled CO_2_ maintained at a low percentage (6%) provides more protection from the adverse effects of brain ischemia than high percentage (9%) CO_2_ ^16^. Of importance, severe hypercapnia, induced by inhaling 15% CO_2_, has been shown to aggravate neurologic injury. Inhaled CO_2_ is more beneficial than low minute ventilation and additional dead space in the reduction of lung inflammation. These results are most likely due to a more homogeneous CO_2_ distribution in the lung parenchyma^17^.

Recently, it has been generally accepted that injury caused by inappropriate MV, including barotraumas, volutrauma and atelectrauma, will likely develop into local inflammatory disequilibrium or even systemic inflammatory reactions which are characterized by cytokine production, neutrophil recruitment and lung edema, resulting in impaired lung function. In animal models of VILI, both high distending volumes, and cyclical airway closure and reopening, have been associated with an increase in lung neutrophil accumulation, as well as increased BAFL levels of inflammatory mediators (TNF-α and IL-8). VILI has been shown to consistently result in the release of inflammatory mediators and cytokines. A significant increase in interleukin (IL)-1, IL-6, and tumor necrosis factor (TNF)-α were observed in BALF after conventional mechanical ventilation over 36 hours in patients with ARDS^18^. However, inhalation of anti-inflammatory mediators such as IL-10 can reduce lung injury^19^.

In the current study, high tidal volume MV markedly increased the production of IL-8 and TNF-α compared to the control group. These findings suggest a central role for IL-8 and TNF-α up-regulation in lung inflammation induced by mechanical stretch. In addition, treatment with hypercapnia significantly decreased the expression of IL-8 and TNF-α, suggesting that hypercapnia has potential anti-inflammatory effects for the treatment of VILI by inhibiting the production of inflammatory cytokines.

Previous studies have found that neutrophils are predominant in the BALF obtained from animals undergoing high tidal volume MV. MPO activity (a peroxidase enzyme that is most abundantly expressed in neutrophil granulocytes) reflects neutrophil infiltration following local tissue or organ injury. Attenuation of MPO activity has been shown to reduce associated pulmonary neutrophil infiltration and improve lung histology in an isolated and perfused rat lung model. The current study showed that high tidal volume ventilation was associated with a significant increase in MPO activity and that HPC treatment improved the effects of VILI. In addition, our results suggest that HPC can reduce lung inflammation by reducing neutrophil infiltration.

Hypercapnic acidosis may itself have an anti-inflammatory effect. Early studies of VILI by Hickling *et al*.^20^ demonstrated that hyperventilation of normal lungs with high airway pressure produces significant lung injury and death. However, if sufficient amounts of inhaled CO2 were administered to achieve normocapnia, injury was less severe, and death was delayed. In vitro, acidosis suppresses TNF release by lipopolysaccharide-stimulated rabbit alveolar macrophages. De Smet *et al*.^21^ reported that hypercapnic acidosis significantly reduced the levels of TNF-α and IL-6 in the lavage and perfusate from unstimulated and lipopolysaccharide (LPS)-stimulated isolated perfused rat lung, indicating that hypercapnia had a protective effect by modulating inflammatory responses^22^. Increasing evidence suggests that hypercapnic acidosis directly inhibits the activation of NF-κB. Intriguingly, this effect of hypercapnic acidosis may be a property of the CO2 rather than its associated acidosis^23^ ^–^ ^26^.

Uchida *et al*.^27^ found that the inflammatory reflex of the vagus nerve can be activated by acetylcholine released from efferent vagal endings. Acetylcholine, combined with alpha7 nicotinic acetylcholine receptors (a7-nAChR), which are located between macrophages, monocytes, lymphocytes and dendritic cell surfaces, block the NF kappa B signaling pathway, inhibiting pro-inflammatory cytokine synthesis and alleviating the inflammatory response. The present study found that the vagus nerve responds to an increase in PaCO_2_ levels with an increase in discharge rate. In addition, an intact vagus nerve pathway is required for the anti-inflammatory action of hypercapnia as shown through vagotomy. These results implicate efferent vagus nerve signaling in the modulation of acute inflammation in the periphery. Vagotomized VILI rats were not affected by hypercapnia as assessed by pulmonary edema (wet-to-dry ratio), BALF inflammatory cytokines TNF-α and IL-8, neutrophil counts, MPO activity and structural damage. In addition, two recent studies have demonstrated that electrical vagus nerve stimulation attenuates VILI induced by injurious tidal volume^28^, and by a two-hit model (hemorrhagic shock followed by MV)^29^.

The present study has several limitations. First, the study was conducted in anesthetized animals, which might have influenced the results. However, the animals studied were anesthetized similarly. Thus, the effect of anesthesia could be counteracted among the different groups. Second, we only observed the effect of right vagal nerve transection in this study. Thus, the effect of left or bilateral vagal nerve transection needs to be further determined. Third, our data suggest that CAP is important as a mechanism of HPC's protective effect on VILI. However, the exact molecular mechanism needs to be investigated.

## Conclusions

The vagus nerve is at least partially responsible for the anti-inflammatory effects of HPC on VILI. These findings will facilitate further investigation of potential therapeutic approaches for VILI and other inflammatory diseases. Our findings, together with previous research, suggest that the cholinergic anti-inflammation pathway may be a mechanism for the attenuation of VILI. Further studies are needed to better understand the complex protective mechanism of HPC on VILI.

## References

[1] Petrucci N, Iacovelli W (2004). Ventilation with lower tidal volumes versus traditional tidal
volumes in adults for acute lung injury and acute respiratory distress
syndrome. Cochrane Database Syst Rev.

[2] Han B, Lodyga M, Liu M (2005). Ventilator-induced lung injury: role of protein-protein
interaction in mechanosensation. Proc Am Thorac Soc.

[3] Brower RG, Matthay MA, Morris A, Schoenfeld D, Thompson BT, Wheeler A, Acute Respiratory Distress Syndrome N (2000). Ventilation with lower tidal volumes as compared with traditional
tidal volumes for acute lung injury and the acute respiratory distress
syndrome. N Engl J Med.

[4] Ni Chonghaile M, Higgins B, Laffey JG (2005). Permissive hypercapnia: role in protective lung ventilatory
strategies. Curr Opin Crit Care.

[5] Laffey JG, Tanaka M, Engelberts D, Luo X, Yuan S, Tanswell AK, Post M, Lindsay T, Kavanagh BP (2000). Therapeutic hypercapnia reduces pulmonary and systemic injury
following in vivo lung reperfusion. Am J Respir Crit Care Med.

[6] Shibata K, Cregg N, Engelberts D, Takeuchi A, Fedorko L, Kavanagh BP (1998). Hypercapnic acidosis may attenuate acute lung injury by
inhibition of endogenous xanthine oxidase. Am J Respir Crit Care Med.

[7] Laffey JG, Honan D, Hopkins N, Hyvelin JM, Boylan JF, McLoughlin P (2004). Hypercapnic acidosis attenuates endotoxin-induced acute lung
injury. Am J Respir Crit Care Med.

[8] Costello J, Higgins B, Contreras M, Chonghaile MN, Hassett P, O'Toole D, Laffey JG (2009). Hypercapnic acidosis attenuates shock and lung injury in early
and prolonged systemic sepsis. Crit Care Med.

[9] Kapetanakis T, Siempos II, Metaxas EI, Kopterides P, Agrogiannis G, Patsouris E, Lazaris AC, Stravodimos KG, Roussos C, Armaganidis A (2011). Metabolic acidosis may be as protective as hypercapnic acidosis
in an ex-vivo model of severe ventilator-induced lung injury: a pilot
study. BMC Anesthesiol.

[10] Contreras M, Ansari B, Curley G, Higgins BD, Hassett P, O'Toole D, Laffey JG (2012). Hypercapnic acidosis attenuates ventilation-induced lung injury
by a nuclear factor-kappaB-dependent mechanism. Crit Care Med.

[11] Borovikova LV, Ivanova S, Zhang M, Yang H, Botchkina GI, Watkins LR, Wang H, Abumrad N, Eaton JW, Tracey KJ (2000). Vagus nerve stimulation attenuates the systemic inflammatory
response to endotoxin. Nature.

[12] Kessler W, Traeger T, Westerholt A, Neher F, Mikulcak M, Muller A, Maier S, Heidecke CD (2006). The vagal nerve as a link between the nervous and immune system
in the instance of polymicrobial sepsis. Langenbecks Arch Surg.

[13] van Westerloo DJ, Giebelen IA, Florquin S, Bruno MJ, Larosa GJ, Ulloa L, Tracey KJ, van der Poll T (2006). The vagus nerve and nicotinic receptors modulate experimental
pancreatitis severity in mice. Gastroenterology.

[14] Li-Sha G, Xing-Xing C, Lian-Pin W, De-Pu Z, Xiao-Wei L, Jia-Feng L, Yue-Chun L (2017). Right cervical vagotomy aggravates viral myocarditis in mice via
the cholinergic anti-inflammatory pathway. Front Pharmacol.

[15] Mihaylova S, Schweighofer H, Hackstein H, Rosengarten B (2014). Effects of anti-inflammatory vagus nerve stimulation in
endotoxemic rats on blood and spleen lymphocyte subsets. Inflamm Res.

[16] Sinclair S, Souders J, Hlastala M (1998). Severity and distribution of ventilator-induced lung injury is
altered by PEEP prone position and respiratory frequency in normal
rabbit. Am J Respir Crit Care Med.

[17] Sinclair SE, Kregenow DA, Starr I, Schimmel C, Lamm WJ, Hlastala MP, Swenson ER (2006). Therapeutic hypercapnia and ventilation-perfusion matching in
acute lung injury: low minute ventilation vs inspired CO2. Chest.

[18] Hotchkiss JR, Blanch L, Murias G, Adams AB, Olson DA, Wangensteen OD, Leo PH, Marini JJ (2000). Effects of decreased respiratory frequency on ventilator-induced
lung injury. Am J Respir Crit Care Med.

[19] Hoegl S, Boost KA, Czerwonka H, Dolfen A, Scheiermann P, Muhl H, Zwissler B, Hofstetter C (2009). Inhaled IL-10 reduces biotrauma and mortality in a model of
ventilator-induced lung injury. Respir Med.

[20] Hickling KG, Henderson SJ, Jackson R (1990). Low mortality associated with low volume pressure limited
ventilation with permissive hypercapnia in severe adult respiratory distress
syndrome. Intensive Care Med.

[21] De Smet HR, Bersten AD, Barr HA, Doyle IR (2007). Hypercapnic acidosis modulates inflammation lung mechanics and
edema in the isolated perfused lung. J Crit Care.

[22] Pinheiro de Oliveira R, Hetzel MP, dos Anjos Silva M, Dallegrave D, Friedman G (2010). Mechanical ventilation with high tidal volume induces
inflammation in patients without lung disease. Crit Care.

[23] Takeshita K, Suzuki Y, Nishio K, Takeuchi O, Toda K, Kudo H, Miyao N, Ishii M, Sato N, Naoki K, Aoki T, Suzuki K, Hiraoka R, Yamaguchi K (2003). Hypercapnic acidosis attenuates endotoxin-induced nuclear
factor-[kappa]B activation. Am J Respir Cell Mol Biol.

[24] Cummins EP, Oliver KM, Lenihan CR, Fitzpatrick SF, Bruning U, Scholz CC, Slattery C, Leonard MO, McLoughlin P, Taylor CT (2010). NF-kappaB links CO2 sensing to innate immunity and inflammation
in mammalian cells. J Immunol.

[25] O'Toole D, Hassett P, Contreras M, Higgins BD, McKeown ST, McAuley DF, O'Brien T, Laffey JG (2009). Hypercapnic acidosis attenuates pulmonary epithelial wound repair
by an NF-kappaB dependent mechanism. Thorax.

[26] Wang N, Gates KL, Trejo H, Favoreto S, Schleimer RP, Sznajder JI, Beitel GJ, Sporn PH (2010). Elevated CO2 selectively inhibits interleukin-6 and tumor
necrosis factor expression and decreases phagocytosis in the
macrophage. FASEB J.

[27] Su X, Lee JW, Matthay ZA, Mednick G, Uchida T, Fang X, Gupta N, Matthay MA (2007). Activation of the alpha7 nAChR reduces acid-induced acute lung
injury in mice and rats. Am J Respir Cell Mol Biol.

[28] dos Santos CC, Shan Y, Akram A, Slutsky AS, Haitsma JJ (2011). Neuroimmune regulation of ventilator-induced lung
injury. Am J Respir Crit Care Med.

[29] Vannucci RC, Towfighi J, Heitjan DF, Brucklacher RM (1995). Carbon dioxide protects the perinatal brain from hypoxic-ischemic
damage: an experimental study in the immature rat. Pediatrics.

